# Commitment and Human Tone: the Difference between Traditional Service and Nursing Care

**DOI:** 10.17533/udea.iee.v37n1e05

**Published:** 2019-01-06

**Authors:** Maryory Guevara Lozano, Ligia Patricia Arroyo Marles, Beatriz Pérez Giraldo, Beatriz Sánchez Herrera

**Affiliations:** 1 Nurse, Masters. Assistant Professor, Universidad de La Sabana, Bogotá (Colombia). Email: maryorygl@unisabana.edu.co Universidad de La Sabana Colombia maryorygl@unisabana.edu.co; 2 Nurse, Specialist. Director of Nursing at Clínica Universidad de La Sabana , Bogotá (Colombia). Email: ligia.arroyo@clinicaunisabana.edu.co Clínica Universidad de La Sabana Colombia ligia.arroyo@clinicaunisabana.edu.co; 3 Nurse, Masters. Associate Professor, Universidad de La Sabana, Bogotá (Colombia). Email: beatriz.perez@unisabana.edu.co Universidad de La Sabana Colombia beatriz.perez@unisabana.edu.co; 4 Nurse, Masters. Professor of High Academic Prestige, Universidad de La Sabana, Bogotá (Colombia). Email: clara.sanchez@unisabana.edu.co Universidad de La Sabana Colombia clara.sanchez@unisabana.edu.co

**Keywords:** nursing care, adaptation, nursing theory, nursing processes, nursing methodology research., atención de enfermería, adaptación, teoría de enfermería, procesos de enfermería, investigación metodológica en enfermería., cuidados de enfermagem, adaptation, teoria de enfermagem, processo de enfermagem, pesquisa metodológica em enfermagem.

## Abstract

**Objective.:**

To describe the transformation of decisive moments that arise within the nurse-patient and family caregiver interaction to turn them into moments of care capable of favoring adaptation.

**Methods.:**

In a high complexity hospital in the city of Bogotá (Colombia), a "nursing methodological research"-type study was conducted. It was developed in five stages: 1) identification of the institutional route of patients and their caregivers and, within it, the moments of encounter with nursing; 2) typical day of the nurse; 3) analysis of the nurse-patient and family caregiver encounters; 4) literature review on how to strengthen the nurse-patient and family caregiver relationship; and 5) proposal to transform decisive moments into moments of care.

**Results.:**

Patients and their family caregivers usually experience six moments of encounter with nursing that include admission, assessment, satisfaction of basic needs, administration of medication, shift change, and discharge; all of them cross-cut by education and communication. Recognition of experiences during moments of encounter allowed transforming them into moments of nursing care.

**Conclusion.:**

The transformation of decisive moments into moments of nursing care to favor adaptation of patients and their family caregivers is consequence of the nursing commitment and human nature expressed in every encounter of the care process.

## Resumen

**Objetivo.:**

Describir la transformación de los momentos de verdad que se dan en la interacción enfermera-paciente y cuidador familiar para convertirlos en momentos de cuidado capaces de favorecer la adaptación.

**Métodos.:**

En una institución hospitalaria de alta complejidad de la ciudad de Bogotá (Colombia) se realizó un estudio del tipo “investigación metodológica en enfermería”, el cual se desarrolló en cinco etapas: 1) identificación de la ruta institucional del paciente y su cuidador familiar y en ella los momentos de encuentro con enfermería; 2) Día típico de la enfermera; 3) Revisión de los encuentros enfermera - paciente y cuidador familiar; 4) Revisión de la literatura sobre cómo fortalecer la relación enfermera - paciente y cuidador familiar; y 5) Propuesta para la transformación de los momentos de verdad en momentos de cuidado.

**Resultados.:**

El paciente y su cuidador familiar tienen generalmente seis momentos de encuentro con enfermería que incluyen el ingreso, la valoración, la atención de necesidades básicas, la administración de medicamentos, el cambio de turno y el egreso. La educación y la comunicación son transversales a ellos. El reconocimiento de experiencias en los momentos de encuentro permitió transformarlos en momentos de cuidado de enfermería.

**Conclusión.:**

La transformación de los momentos de verdad en momentos de cuidado de enfermería para favorecer la adaptación del paciente y de su cuidador familiar son una consecuencia del compromiso y del tono humano de enfermería expresado en cada uno de los encuentros del proceso de atención.

## Resumo

**Objetivo.:**

Descrever a transformação dos momentos de verdade que se dá na interação enfermeira-paciente e cuidador familiar para convertê-los em momentos de cuidado capazes de favorecer a adaptação.

**Métodos.:**

Em uma instituição hospitalar de alta complexidade da cidade de Bogotá (Colômbia) se realizou um estudo do tipo “investigação metodológica em enfermagem”, a qual se desenvolveu em cinco etapas: 1) identificação da rota institucional do paciente e seu cuidador familiar e nela os momentos de encontro com enfermagem; 2) Dia típico da enfermagem; 3) Revisão dos encontros enfermeira - paciente e cuidador familiar; 4) Revisão da literatura sobre como fortalecer a relação enfermeira - paciente e cuidador familiar; y 5) Proposta para a transformação dos momentos de verdade em momentos de cuidado.

**Resultados.:**

O paciente e seu cuidador familiar, têm geralmente seis momentos de encontro com enfermagem que incluem o ingresso, a valorização, a atenção de necessidades básicas, a administração de medicamentos, o câmbio de plantão e o egresso. A educação e a comunicação são transversais a eles. O reconhecimento de experiências nos momentos de encontro permitiu transformá-los em momentos de cuidado de enfermagem.

**Conclusão.:**

A transformação dos momentos de verdade em momentos de cuidado de enfermagem para favorecer a adaptação do paciente e do seu cuidador familiar sao uma consequência do compromisso e do tom humano de enfermagem expressado em cada um dos encontros do processo de atenção.

## Introduction

The nursing care process must guarantee to patients and their family caregivers a practice of safe care that is technically sound, objective, and subjectively reliable, detectable and, consequently, capable of improving.(1) This is why daily there is increased use of guides and protocols to orient care work in diverse contexts. However, these tools can hardly guarantee adequate care, if they are not imprinted with the art of caring.(2) Although health institutions seek standardized care that is measurable and focused on productivity, the nursing practice experiences daily the conflict of a philosophical humanist view, embodied in its conceptual models and theories. To be implemented, these could require greater nurse-patient and family caregiver interaction, and which contrast with the reality of the financial costs of institutions pressured into being increasingly efficient and, consequently, more impersonal. 

Within this context, going from performing activities and procedures to looking to respond to the paradigm of caring for the health experiences of individuals, implies for Nursing to dare go beyond the known environments and again question their reason for being, to achieve equilibrium in the practice with positive impact on caring for patients and their family caregivers, while responding to the demands of the context. 

In the *Universidad de La Sabana Clinic,* it has been understood that in spite of the difficulties inherent to the system to provide authentic nursing care, it is necessary to think about how to develop a significant practice for patients and their family caregivers, as well as for the nursing profession. This work emerges from these reflections and seeks to describe the transformation of the decisive moments taking place in the nurse-patient and family caregiver interaction in this institution to convert them into moments of care capable of favoring the adaptation. The work permits improving the nursing practice(3) in favor of patients and their family caregivers, within a specific context that experiences the tensions of a health system that privileges economic criteria. It responds to the reason for being of Nursing and heeds the trends and conceptual guidelines in effect in the professional discipline(4) and provides knowledge to a current global problem. 

## Methods

This work is framed as Nursing methodology research,(5) conducted between 2016 and 2018, as part of strengthening the teaching-care alliance between the Universidad de La Sabana Clinic and the Faculty of Nursing and Rehabilitation at the same institution. The study was approved by the pertinent instances within the Institution and developed within the framework of international ethical and environmental principles. The process implied the following stages:

*(i) Identification and description of the institutional route of patients and their family caregivers and of the moments of encounter with Nursing*. For this purpose, a Nursing group was assigned during two months to follow the movements of randomly selected patients and their family caregivers, through prior consent, keeping a field diary. The route traced was analyzed to recognize in them the common pattern of encounters among patients and their family caregivers, and Nursing, individually or collectively to describe when, how, where and for how much time these took place and the need or service offer in each case. *(ii)* Complementing the aforementioned, another group of observers *analyzed the typical day of nurses;* and revised *(iii) encounters with patients and their family caregivers*, within their care route. The outcomes of the routes were contrasted, evidencing some decisive moments occurring since the patients and their family caregivers were admitted into the institution, were expressed during the care process and continued until the moment of discharge. The common characteristics were then revised; following, for this purpose, the analysis parameters for this type of interaction.(6) The study considered, specifically as qualities or attributes, the type and form of communication, knowledge, tone of the relationship, exercise of roles, and guaranty of necessary resources. It sought to understand care in terms of its meaning and repercussions for the parts implied. It was verified if learning occurred during this process, including the pedagogic methods and the way of assessing care from the perspective of those implied. 

*(iv)* Upon identifying the encounters among patients and their family caregivers, and nurses, a literature review was conducted to answer the question: What does the global literature say about how to strengthen the moments of encounter between the nurse and the subject of care? The search criteria established the following: inclusion of literature reviews, case studies and controls, experimental studies, systematic reviews, and clinical guides based on evidence, as long as they were in indexed journals. The search included the following databases: PubMed, ScienceDirect, Ovid Nursing, CINALH, SciELO, and Bireme. In addition, the lists of bibliographic references of the studies selected were revised. The window of observation was 2003 to 2016, in English, Spanish, and Portuguese, with the following formula [Decisive moment OR Openness OR Honesty OR Presence OR Sensitivity OR Sensitive Care OR Sensitive Nursing] AND [Nursing care, OR Adaptation OR Nursing Processes OR, Patient Care Planning]. Data organization and tabulation considered the reference, objective, methodology, results, and relevant aspects. Critical analysis was performed of the findings with interpretation of the content grouped in integrative manner to interpret from this the recommendations for the nursing practice. The search was complemented with a revision of the best practices in the service available in “*Advisory Board*”(7) and the Nursing Outcomes Classification (NOC) was revised to propose the indicators. *(v)* Lastly, based on the diagnosis and the review, a proposal was made to *strengthen the most frequent decisive moments among patients and their family caregivers and nurses*. This involved specifying the concepts of nursing care and moment of nursing care for the institution. 

## Results

The route of patients and their family caregivers goes from the programmed or emergency admission to the discharge from the hospital. In it, the experience is shared between patients and their family caregivers with various moments of encounter with nurses that include admission, general assessment and the pain permanent, care of basic needs, change of shift, administration of medications, and discharge. Each of those moments include education and communication activities among patients and their family caregivers, and nurses. Eventually, other encounters take place responding to specialized care or procedures.

The moment of encounter among patients and their family caregivers, and nurses was defined as a decisive moment of nursing care in the institution. The encounters were heterogeneous in the amount and content of communication and, although the exchange tends to be friendly, important differences were noted in the tone of the relationship. The principal roles of Nursing during the encounters were those of caring and educating, although on some opportunities other roles emerged, like advisory, advocacy, and the administrative role. During the encounters, there was much logistics activity by nurses to guarantee the necessary resources of the environment for patient care. For the patients, being cared for meant being identified as persons, having timely care, receiving treatment, and receiving a response to their concerns. For the family caregivers, care meant their loved ones were properly cared for in timely and friendly manner; in general, they did not identify themselves as subjects of care. For nurses, care was associated with the guaranty of safety, diminished risk, suitability, kindness and recognition of patients and their family caregivers as people. In no case was care seen as an opportunity for interpersonal growth. The educational activity was frequent; most of the cases had no didactic aids other than an illustrative sheet or booklet. The comprehensive evaluation of care identified as a priority challenge of the Nursing direction. 

Literature showed that to strengthen the moments of encounter between a nurse and the subject of care, it is necessary to prioritize the “authentic presence”. This presence has been approached under different perspectives, with the following attributes: 1) Nursing devotion in an intersubjective relationship, where mutual openness and unconditional love is experienced along with a sense of comfort when present; 2) availability of nurses to respond to the person’s needs; 3) presence that supposes concentrating on the other person’s concerns, leaving aside the nurse’s own concerns; 4) presence in a nursing intervention, like the skill to understand what others are feeling or experiencing; 5) presence amid the time of caring for the person that must be cared for; 6) presence as the nurse’s experience of devotion in an authentic presence capable of transforming what is hidden or saved to generate hope, expansion of awareness, and care; 7) presence as a direct goal that responds to a call and has the intention of giving it all looking for a path of help; and 8) presence as something intangible, mutual, and recognized through tact, tone, or sight.(8) 

This “authentic presence” is not a mechanism to obtain information, nor is it a form of changing or directing the person’s health experience, or of having an empathic behavior. Authentic presence is a way of being with people during significant situations in their lives, of understanding their context. It is a subject-to-subject relationship that honors each of the changes of the other individual’s reality in their uniqueness in the midst of their health situation and which demands commitment and devotion to make sure the other person is the center.(9) Nursing narratives have been an important aid to understand the meaning and form of being present. From the physical, it is being close to another when closeness generates safety and calm to the subject of care. The total presence is unique, requires empathy, permits care and propitiates innovation. Presence can also be transcendent, of spiritual nature, and it implies connection and synchrony with something or someone and is associated with wellbeing, peace, and comfort, being the most powerful form of restoring the whole person to heal them; it demands besides the technical-scientific, knowing when and how to act.(10) Authentic presence in nursing care is one of the most powerful interventions of caring for patients and their family caregivers, especially during moments of marked vulnerability, and which can impact positively satisfaction and transform the lives of those implied.(11) Having authentic presence in the encounter of nurses with patients and their family caregivers means being with them, observing, listening, learning, and supporting the experience of living or dying. It is necessary for nurses to be attentive to the situation, active, dedicated, sensitive to the context, isolate their prejudices and be empathic.(11) “Being there” is a concrete form of including the spiritual dimension in care and that permits resignifying the decisive moment. This implies getting support from observation and intuition and being able to recognize the call. “Being there” implies a loving presence, which takes care of detail and seeks peace; it is listening with especial attention, it is respecting and becoming interested in the feelings expressed. “Being there” is speaking to provide valuable help that has to do with informing, guiding, or supporting, and it is having physical contact to express compassion and companionship.(12)

Understanding what characterizes that during a decisive moment, nursing care is perceived, from the perspective of patients or of their family caregivers, as recognized as unique, feeling that their interests and tastes, their values and beliefs are kept in mind. This supposes making a connection, knowing how to recognize the details, and understanding how people want to be cared for; due to this, nursing should be attentive to the experiences of the individuals and respond in anticipated manner or when said individuals require it. The transformation of a decisive moment into a moment of care requires courage, curiosity, collaboration, consideration, commitment, celebration of positive aspects and especially, emotional connection.(13) The perception of affect and demonstration of sincerity by Nursing is recognized as a mechanism to transform a moment of nurse-patient encounter into a moment of care.(14)

**Figure 1:**
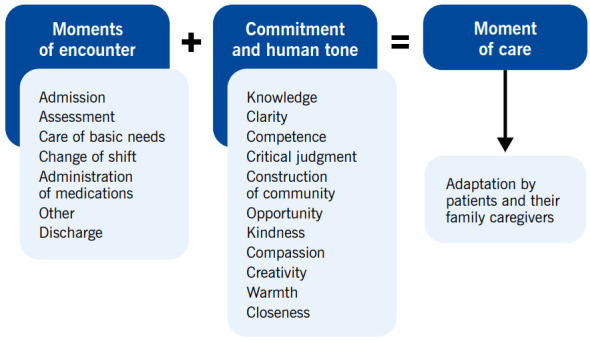
Transformation of moments of encounter into moments of nursing care in the Universidad de La Sabana Clinic

Retaking the routes and encounters of patients and their family caregivers with Nursing, in the Universidad de La Sabana Clinic, in light of the experiences and the literature review, the necessary conditions were established to favor adaptation during the experience in the institution. These were united into two big categories: commitment that must always be supported in the best evidence available and is associated with the science of caring, and the human tone, or the attribute associated with the caregiver's own condition of being and being present and which is related with the art of caring. It is the sum of these categories that manages for a moment of encounter of patients and their family caregivers, with nurses, to become a moment of care; a moment in which Nursing makes the difference for them, in spite of their vulnerability, to feel “at home”, in what for Nursing in the institution has been denominated “adapted”. This moment of care guarantees safety, seeks comfort and wellbeing, respects dignity, and aims to strengthen their autonomy. This change implies nursing autonomy; touches the transcendent sense of people and enhances them in their roles as receptors or caregivers (Figure 1).

## Discussion

This work, because of its humanistic nature that considers the subject of care, patient, and family caregiver as the center of nursing care, is similar to various conceptual proposals exposed by Ann Mariner,(15) which include that by Ann Boykin and Savina Schoenhofer, known as the *Care Model for Transforming Practice*; Jean Watson’s *Human Care*; and Callista Roy’s with the *Conceptual Adaptation Model* that recognizes the wellbeing of the human person as central. Upon revising the successful experiences of other institutions to determine how to improve nursing care, we describe the importance of defining the route.(16) The same has occurred when seeking to improve quality or when requiring implementation of a conceptual systematic model. In this case, establishing the route for patients and their family caregivers and within such, the moments of encounter with Nursing were the key to recognizing and transforming decisive moments into moments of nursing care that propitiate adaptation. 

The "decisive moment" is understood herein as the time during which the user comes into direct contact with the health care service and, based on this contact, formulates an opinion on the quality of said service.(17) This moment, as evidenced, occurs through the encounter of patients and family caregivers with nurses; in it, as in other cases, in the hospital care route, where the concept of authentic presence, can be analyzed in depth.(18) This study describes six key moments and two cross-sectional moments, admitting that others can exist. These are the moments of admission, assessment, care of basic needs, administration of medications, change of shift, and discharge. Education and communication are cross-sectional. No studies or models were found to admit together these moments of encounter or their transformation into moments of nursing care. 

The moment of admission has been described as a key moment in the perception of satisfaction by patients and their family caregivers. It requires empathy and direct communication; prioritizing aspects, like pain and the patient’s condition; revision of their medication conciliation; bearing in mind context, age, wait period and availability Nursing human talent to propose a care plan guided by goals and accompanied by educational strategies, which leads to modifying positively the experience of patients and their family caregivers.(19) Regarding the assessment by Nursing, work has been carried out on measuring adherence to protocols as base to improve the quality of patient-centered care; tools to evaluate specific aspects, like pain and suffering; unpleasant symptoms and recognition of the strengths and needs to favor the adaptation.(20) Care of basic needs, as a central function of the nursing work, includes hygiene, comfort, feeding, moving, dressing, and going to the bathroom. This moment of encounter considers and seeks to maintain the level of independence and satisfy the requirements related with daily life activities.(21)

The administration of medications is perhaps the moment with the most solid evidence in the literature. Its findings have centered on the error committed during this process and on the search for safety_,_ strategies, and technological support that reinforce safe behaviors, along with specialized accompaniment, and continuous formation on the theme, especially indicating that combined strategies are the most successful.(22) However, little has been differentiated between safe traditional care and nursing care during this moment of care, where it is necessary to transcend a correct scheme to arrive, as has been indicated, at a comprehensive and transcendent care scheme. The change of shift in Nursing has been recognized as a key moment to guarantee the continuity and safety of patients and their family caregivers and, although its evidence is still weak, the best practices suggest using guides, incorporating patients and their caregivers, and revising convenient shifts for the staff under care transference schemes.(23) The moment of discharge has greater conceptual clarity and evidence that supports its necessity as a factor to prevent readmissions and complications. However, evidence against the forms of performing it and its effectiveness are still quite weak.(24)

In synthesis, it is possible to state that, in spite of the growing documentation during each of these moments, the relationship among patients and their family caregivers, with nurses is scarcely mentioned. The studies reviewed reflect that the decisive moments are not always identified as susceptible to transformation to convert them into moments of nursing care, as proposed in this work. It is necessary to revise the scenario desired by Nursing in its function as caregiver to make this transformation and manage to care for the experience of patients and their family caregivers(25) within the institutional environment to, therein, guarantee its adaptation. 

This study concludes that the transformation of decisive moments into moments of nursing care to favor the adaptation of patients and their family caregivers, in the Universidad de La Sabana Clinic, is consequential of the commitment and human tone of Nursing expressed during each of the encounters of the care process. Defining this route and focusing the nurse’s work on the experience of the subject of care, in this case patients and family caregivers, to care for it, as proposed by the Nursing paradigm, is a different contribution that can permit greater social impact on the professional field and a more sound epistemological and ontological construction from the disciplinary perspective. 

Recognizing and summarizing the characteristics of care necessary to modify the moments of encounter or decisive moments into true moments of care, require the application of all the patterns of nursing knowledge and can be specified in the relevance of assuming and demonstrating responsibility and respecting, without amendment, the dignity of the human person. In addition to following the care route, identifying decisive moments in the relationship of patients and family caregivers, with nurses has added to a strategy that accepts and articulates the science and art of caring, and permits making the difference between traditional service and nursing care. 
